# Prediction of Unplanned Hospitalizations in Older Patients Treated with Chemotherapy

**DOI:** 10.3390/cancers13061437

**Published:** 2021-03-22

**Authors:** Jaime Feliu, Enrique Espinosa, Laura Basterretxea, Irene Paredero, Elisenda Llabrés, Beatriz Jiménez-Munárriz, Beatriz Losada, Alvaro Pinto, Ana Belén Custodio, María del Mar Muñoz, Jeniffer Gómez-Mediavilla, María Dolores Torregrosa, Patricia Cruz, Oliver Higuera, María José Molina-Garrido

**Affiliations:** 1 Oncology Department, Hospital Universitario La Paz. IDIPAZ, Cátedra UAM-AMGEN, Centro de Investigación Biomédica en Red de Cáncer, 28046 Madrid, Spain; eespinosa@salud.madrid.org (E.E.); alvaro.pinto@salud.madrid.org (A.P.); ana.custodio@salud.madrid.org (A.B.C.); patricia.cruz@salud.madrid.org (P.C.); oliver.higuera@salud.madrid.org (O.H.); 2 Oncology Department, Hospital Universitario de Donostia, 20014 Donostia, Spain; laura.basterrecheabadiola@osakidetza.eus (L.B.); jgomez@onkologikoa.org (J.G.-M.); 3 Oncology Department, Hospital Universitario Dr. Peset, 46017 Valencia, Spain; irene.paredero@hospitalprovincial.es (I.P.); m.dolores.torregrosa@uv.es (M.D.T.); 4 Oncology Department, Hospital Universitario Insular de Gran Canarias, 35016 Las Palmas, Spain; ellaval@gobiernodecanarias.org; 5 Oncology Department, Centro Integral Oncológico Clara Campal, 28050 Madrid, Spain; bjimenez@hmhospitales.com; 6 Oncology Department, Hospital Universitario de Fuenlabrada, 28942 Fuenlabrada, Spain; beatriz.losada@salud.madrid.org; 7 Oncology Department, Hospital Virgen de la Luz, 16002 Cuenca, Spain; madelms@sescam.jccm.es (M.d.M.M.); mjmolinag@sescam.jccm.es (M.J.M.-G.)

**Keywords:** older patient, unplanned hospitalizations, risk score, geriatric assessment, chemotherapy

## Abstract

**Simple Summary:**

Unplanned hospitalizations (UHs) are common among elderly patients with cancer who receive chemotherapy. This fact decreases quality of life or performance status and increases health costs. Our objective was to determine predictive factors for UH in this population. A score based on six variables taken from geriatric assessment and chemotherapy characteristics was developed in a series of 493 elderly patients receiving chemotherapy. The use of this score may reliably identify patients at risk for UH, thus helping to plan treatment, implement adaptive measures, and set up a close follow-up schedule.

**Abstract:**

Purpose: To determine the incidence of unplanned hospitalization (UH) and to identify risk factors for UH in elderly patients with cancer who start chemotherapy. Methods: In all, 493 patients over 70 years starting new chemotherapy regimens were prospectively included. A pre-chemotherapy geriatric assessment was performed, and tumor and treatment variables were collected. The association between these factors and UH was examined by using multivariable logistic regression. Score points were assigned to each risk factor. Results: During the first 6 months of treatment, 37% of patients had at least one episode of UH. Risk factors were the use of combination chemotherapy at standard doses, a MAX2 index ≥1, a Charlson comorbidity score ≥2, albumin level <3.5 g/dL, falls in the past 6 months ≥1, and weight loss >5%. Three risk groups for UH were established according to the score in all patients: 0–1: 17.5%; 2: 34%; and 3–7: 57% (*p* < 0.001). The area under receiver operation characteristic (ROC) curve was 0.72 (95% CI: 0.67–0.77). Conclusion: This simple tool can help to reduce the incidence of UH in elderly patients with cancer who are scheduled to initiate chemotherapy treatment.

## 1. Introduction

Chemotherapy may benefit quality of life in elderly patients with cancer through increased survival and symptom relief [1,2], but aging also favors the appearance of severe toxicities and other complications such as the worsening of preexisting conditions [3]. An undesirable consequence of antineoplastic treatments in the elderly is the increase in hospital admissions, since it may have a significant impact on the patient’s quality of life, causing an irreversible decline in functional capacity and loss of independence and leading to institutionalization in many cases [4,5]. Hospitalization is a costly [6] and distressing experience for patients with cancer and their families. Moreover, as elderly people usually prioritize quality over quantity of life, hospitalization may interfere with patients’ stated preferences.

Up to 35% of patients with cancer have an unplanned hospitalization (UH) during the first year following diagnosis. This percentage rises to 42.5% in those older than 75 [7].

Chemotherapy-derived toxicity accounts for 30–40% of hospitalization events in cancer patients [8], and an estimated 9% of patients receiving chemotherapy need hospitalization for that reason [9]. UH in the elderly can be the result of toxicity, worsening of cancer symptoms or worsening of comorbidities as a consequence of cancer or its treatment. A recent study including 8451 cancer patients aged ≥70 reported an incidence of UH of 20% in the next three months following the initiation of therapy [10].

Few studies have focused on older adults with cancer, and risk factors associated with UH are not well defined. Critical factors in younger adults are comorbidity [7,8], as well as renal and bone marrow function [8]. Additional factors such as age and Eastern Cooperative Oncology Group (ECOG) performance status have been described in older patients [11]. Some studies have assessed the value of the geriatric assessment (GA) or its components to predict UH, with conflicting results. One study could not correlate GA with the risk of UH [12], whereas others have identified some related factors, such as dependency in instrumental activities of daily living (IADL) or in activities of daily living (ADL), malnutrition, presence of comorbidities, abnormal G8 [10], or reduced gait speed [13].

Previous studies in this setting included older patients receiving either chemotherapy or just palliative supportive care [7,10] or were focused on hematologic malignancies [13]. The identification of factors predicting UH in the older patients receiving chemotherapy could be used to set up adaptive or aggressive supportive measures around the time of chemotherapy initiation to prevent UH. It would also select patients in whom the chemotherapy schemes should be modified to reduce the risk of toxicity and would improve the chemotherapy informed-consent process. Finally, it could contribute to improve quality of life and quality of care and to reduce health care costs (provided this leads to directed interventions).

The objective of this study was to determine the incidence of UH and associated risk factors in older patients (age ≥ 70 years) with cancer who start chemotherapy.

## 2. Results

### 2.1. Patient and Tumor Characteristics

Among the 551 patients who completed baseline assessment, three patients moved to another center after the first cycle of treatment, two died from rapidly progressing cancer prior to treatment, two patients withdrew consent early, and four were treated with targeted therapies without chemotherapy and were excluded from the analysis. Information about UH was not provided by treating centers in 47 patients, so the series finally included 493 patients.

Baseline patient characteristics, including demographics, GA, chemotherapy, and laboratory findings, are shown in Table 1. Median age was 77 years (range 70–92, 35% were ≥80 years); most patients had a good performance status with ECOG ≤ 1 (87%), staging was I–III (42%) and IV (58%). The most common tumor types were gastrointestinal (52%), genitourinary (13%), lung (12%), and breast (6%). Fifty-nine percent of patients received polychemotherapy, 44% received standard doses of chemotherapy. Chemotherapy was administered in the adjuvant or neoadjuvant setting in 37% of patients, as first line in 54%, and as second or subsequent line in 9%. Primary prophylaxis with granulocyte colony-stimulating factor growth factors was used in 14% of patients. Thirty-two percent of patients received concurrent chemotherapy and radiation therapy.

The GA detected that 15% of patients lived alone and 75% had less than an associate/bachelor’s degree. According to the Charlson comorbidity index, 37% of patients had comorbidity greater than or equal to grade 2, and 22% and 44% were dependent on ADL and IADL scales, respectively. Impairment of cognitive function was detected in 11% of the patients. Thirteen percent had a low body mass index of less than 22 kg/m^2^, and 50% had a score ≥3 according to the Vulnerable Elders Survey−13.

### 2.2. Incidence and Reasons of Unplanned Hospitalizations

**Figure 1:**
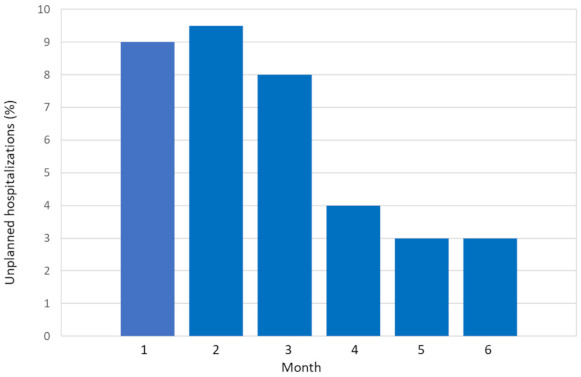
Percentage of unplanned hospitalizations by month.

After a follow-up of 6 months, 184/493 patients (37%) had had at least one UH. There was one episode in 72% (132/184), two in 21% (39/184), three in 3% (12/184), and four in 0.5% (1/184). Most patients (133/184, 72%) had the first UH in the first 3 months (Figure 1). Table 2 outlines the causes of UH, the most common being for cancer-related reasons (29%), chemotherapy toxicity (19%), and infections (either in the lungs, 16%, or somewhere else, 12%).

### 2.3. Predictive Variables Associated with Unplanned Hospitalizations in the First 6 Months

In the univariable analysis, we identified 11 factors that were associated with UH (Table 3). The risk factors associated with 6-month UH in univariable analysis (*p* < 0.05) and variables deemed to be of clinical importance (i.e., age and tumor subtype) were included in the multivariable analysis. In the multivariable model, only six independent variables were directly associated with a higher risk of UH (Table 4): the administration of standard-dose combination chemotherapy, a MAX2 index ≥1, a Charlson comorbidity score ≥2, albumin level <3.5 g/dL, falls in the past 6 months ≥1, and unintended weight loss >5%.

**Figure 2:**
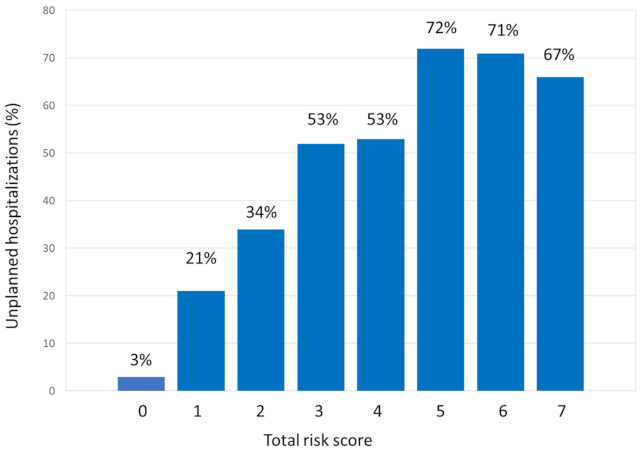
Ability of risk score to predict unplanned hospitalizations in the next 6 months.

The risk score was applied to each patient, and patients were classified into three categories on the basis of the risk of UH: low risk (0–1 points: 17.5% 6-month UH rate), intermediate risk (2 points: 34% 6-month UH rate), and high risk (3–7 points: 57% 6-month UH rate). The proportion of patients classified as low, intermediate, or high risk were 32%, 30%, and 38%, respectively (Figure 2). There was a significant difference in the mortality within the first 6 months among the risk groups (*p* < 0.001).

The area under receiver operation characteristic (ROC) curve was 0.72 (95% CI: 0.67–0.77) (Supplementary Figure S1). Exploratory analyses were performed to calculate the model performance of the total risk score according to stage (localized 0.71 and disseminated 0.73), and for each tumor type: (GI 0.70, GU 0.78, breast 0.68, lung 0.61, another cancer 0.89). Calibration of the final model was assessed by the Hosmer–Lemeshow goodness of fit test. A *p*-value of 0.49 (95% CI, 0.45–0.53), suggests that the model is accurate.

## 3. Discussion

Over one-third of patients in our series had a UH in the 6 months following the start of chemotherapy. This complication is particularly relevant in the elderly because of its impact on quality of life and of the risk that hospitalization leads to an irreversible decline in functional capacity and loss of independence. Hospitalization also increases health costs, estimated at 48% of the cost of medical care of patients with advanced tumors [14]. The identification of risk factors for UH could lead to the implementation of preventive measures.

The factors predicting UH in our population can be easily obtained as they are gathered in daily practice. Some of them are related to the treatment itself, whereas others are part of the GA: nutritional status (albumin and weight loss), performance status (falls), and comorbidity (Charlson index). All these factors relate with some of the conditions known to cause UH in older patients: chemotherapy toxicity, comorbidity, and frailty.

The risk of developing grade 3–4 toxicity has been previously correlated with standard-dose combination chemotherapy [8,15,16,17] and treatment aggressiveness (MAX2 index) [17]. Falls have also been identified as a risk factor for increased toxicity [16]. On the other hand, some studies have found that comorbidity [8,9,10,11,18], chemotherapy, poor nutritional status [10], and low albumin level [8,19] increase the risk of UH. Serum albumin decreases 15–20% with age, even more in patients with poor nutrition. This may result in an increase in the free fraction of the drug in plasma, as described with cisplatin, etoposide, taxanes, or methotrexate [20], thus increasing adverse events.

Unlike other studies [10,13,17], we did not find an association of UH with poor ECOG performance status, ADL, or gait speed in the multivariate analysis. However, the history of falls was significant. This factor relates not only to the functional status but also to the risk of chemotherapy toxicity [16]. The role of age is controversial. We and others [10] did not find a direct correlation, although two studies performed in the community population and in patients with colon cancer, found a correlation of UH with advanced age [17,21]. Finally, one study reported less risk of UH in the elderly, probably due to the use of less-aggressive treatment [8].

Of note, 87% of patients had an ECOG performance status ≤1. This is higher than that reported in other studies [10] and could have biased the results. The good condition in our patients derives from the fact that only those amenable for chemotherapy were included, whereas other studies also included patients receiving just supportive care [10].

To our knowledge, this is the first prognostic score to address the risk of UH in older patients with cancer who are going to initiate chemotherapy. The score is simple, easy to implement, and reliable in identifying groups of patients with different risk of UH. Although three risk groups were established, risk increases with score also within the group. Therefore, in the high-risk group (score 3–7 points) there is a 57% 6-month UH rate, but this percentage rises to 71% in patients with 5–7 points.

The identification of patients at increased risk for UH may guide specific intervention, for instance, the use of less toxic schemes, reduced doses, or single-agent therapy, particularly in patients receiving therapy with palliative intent. However, if therapy is used with curative intent, careful consideration should be taken as dose modification could reduce efficacy. Likewise, measures could be initiated to improve the nutritional and functional status of older patients, although the randomized study GAIN (Geriatric assessment-driven intervention) showed that such measures only reduce drug toxicity but not UH or the number of visits to the emergency department [22]. Supportive care and close monitoring should be established in high-risk patients.

The strengths of this study are its multicenter design and the development of a score that is simple and easy to use. Regarding its limitations, (1) patients with hematological malignancies were not included, so the utility in this population remains unknown; (2) there is no external validation, so we do not know whether the score could be used in a different population; (3) the score predicts UH in the first 6 months of therapy but not beyond that time. However, most UHs happen in the first 3 months [9]; (4) the score was developed in older patients receiving chemotherapy, so we do not know its validity in those receiving other treatment such as immunotherapy or targeted therapies.

The present study indicates that a score based on six variables taken from geriatric assessment and chemotherapy characteristics may reliably identify older patients at risk for UH. The use of this simple tool could help to plan treatment and establish interventions and recommendations to decrease risks in this population.

## 4. Patients and Methods

### 4.1. Patients

This prospective and multicenter study was performed in eight hospitals in Spain between February 2014 and June 2018. The main objective was to assess factors predicting the occurrence of grade 3–4 toxicity in older patients who received chemotherapy [14]. Patients aged ≥70 years with histological or cytological confirmation of malignancy in any stage were included. Other inclusion criteria were as follows: (1) initiation of a new chemotherapy regimen, (2) the ability to read Spanish (questionnaires for geriatric assessment were in Spanish), and (3) outpatient condition. All patients provided written informed consent to participate in the study. The study was approved by the institutional review board at each participating institution.

Here, we present a sub-study focusing on the incidence of UH and associated risk factors in older patients with a solid cancer, within 6 months from the start of chemotherapy. A UH was defined as any inpatient admission to an acute care hospital that began after the day of starting chemotherapy and that could not be foreseen. It may occur on a non-emergency or an emergency basis [23]. Data on unplanned hospitalizations were collected retrospectively.

### 4.2. Study Schema

Clinical staging was performed according to routine clinical practice depending on each cancer type. Patients completed GA before the start of chemotherapy. This assessment included a standardized evaluation of their social support and comorbidity, as well as their functional, cognitive, psychological, and nutritional status (Table S1). The questionnaire was delivered by a research nurse; one part was completed by the patient and another one by the health professional. The latter included the following items: comorbidities (collected using the Charlson comorbidity index) [24], Eastern Cooperative Oncology Group performance status [25], Short Physical Performance Battery (gait speed, chair rise, and progressive Romberg) [26,27], the body mass index, and the Pfeiffer test [28]. The Pfeiffer test or Short Portable Mental Status Questionnaire (SPMS) is an easily administered, validated, 10-item screen for cognitive impairment that has been tested in clinical and institution-based samples. Test–retest reliability was ≥0.82, and the agreement between the SPMS and clinical diagnosis for intact cognition or mild impairment was 82%. It has been established that three or more mistakes suggest cognitive impairment and advise a thorough study of cognitive function, always taking cultural and study levels into consideration. The patient part consisted of self-reported measures of functional status: activities of daily living (ADL) [29], instrumental activities of daily living (IADL) [30], number of falls in the last 6 months, nutrition, medications, psychological state [31], social support/function [32,33], and ability to take medications unassisted. A member of the healthcare team assisted those who needed help with completing the questionnaires.

The following variables were collected in addition to the results of the GA: age, gender, marital status, household composition, education, cancer subtype and stage, and selected blood tests obtained before treatment (hemoglobin, white blood cell count, platelets, creatinine, albumin, liver function test, and creatinine clearance [34]). Data regarding the type of chemotherapy, line of treatment, and use of granulocyte-growth factors were also recorded.

The risk of chemotherapy-induced toxicity for every chemotherapy regimen administered was estimated with the MAX2 index toxicity [35]. The MAX2 index summarizes the overall risk of severe chemotherapy-induced toxicity based on an average study using data from published clinical trials. Briefly, the MAX2 index is the average of the highest frequency of both grade 4 hematologic toxicity and grade 3/4 non-hematologic toxicity, with higher scores indicating higher risk of toxicity. It is reproducible across cancer types and studies, and it is sensitive to toxicity differences among chemotherapy regimens.

Patients were prospectively followed until the end of the chemotherapy course. UHs occurring in the 6 months following the start of treatment were recorded.

### 4.3. Statistical Analysis

The primary outcome was the occurrence of UH within 6 months after the start of treatment. Descriptive statistics characterizing patient groups were provided. The X^2^ test was used to examine the association between UH and categorical variables and independent t tests for continuous variables.

We performed a correlation assessment using the Spearman’s rho test as appropriate for categorical variables. Multicollinearity between variables was defined as a rho test value ≥0.50. An evaluation of UH predictors was performed by using logistic regression. Univariate models were first fitted for all prognostic factors. Significant variables at the 5% level were selected for inclusion in the multivariable model. Odds ratios (ORs) were reported with their 95% CIs. *p* < 0.05 was considered statistically significant for all comparisons. The optimal cut-point for the continuous variables was determined using the Youden index, and the categorical variables were dichotomized according to clinically relevant cutoffs. The amount of accounted variance was determinate with the Nagelkerke correlation coefficient (R^2^). Model calibration and discrimination were assessed by the Hosmer–Lameshow test and the area under the receiver operating characteristic (ROC) curve [36,37]. For the development of the score, each factor was assigned a particular score based on its β coefficient. The β coefficient for each risk factor was divided by the lowest β coefficient and rounded to the nearest whole number [38,39]. The risk score was then applied to each patient. The sample was divided into three risk strata (low, medium, and high risk of UH) on the basis of approximate tertiles of risk score. We compared UH within the first 6 months among the risk groups by chi square testing. We used the bootstrap method (1000 repetitions) for internal validation of the risk score. Bootstrap validation is a method of random resampling from a given set of samples to simulate the effect of drawing samples from the same population.

Analyses were carried out by with SPSS software (version 18; SPSS, Chicago, IL, USA).

## 5. Conclusions

A risk score to predict UH in elderly patients receiving chemotherapy was developed. The score combines data from the GA and the characteristics of chemotherapy. This tool may help in treatment planning and in the implementation of measures aimed at improving the care of our patients and reducing UH.

## Figures and Tables

**Table 1:** Patient characteristics (*n* = 493).

Characteristic	Patients, *n* (%)
Age	
70–74	169 (34%)
75–79	152 (31%)
≥80	172 (35%)
Sex	
Male	310 (63%)
Female	183 (37%)
Tumor site	
Gastrointestinal	256 (52)
Genitourinary	64 (13)
Lung	59 (12)
Breast	30 (6)
Gynecologic	15 (3)
Other	69 (14)
Metastatic status	
M0	209 (42%)
M1	284 (58%)
ECOG PS	
0	126 (25%)
1	304 (62%)
2	63 (13%)
IADL	
8	217 (44%)
≤7	276 (56%)
ADL	
6	387 (79%)
≤5	106 (21%)
Number of falls in the past 6 months	
None	409 (83%)
≥1	85 (17%)
Charlson comorbidity score	
0	166 (34%)
1	142 (29%)
≥2	185 (37%)
VES-13	
0–2	171 (35%)
≥3	322 (65%)
Chemotherapy	
Standard therapy	215 (44%)
Reduced therapy or monotherapy	278 (56%)
MAX2 index	
0	151 (31%)
1	292 (59%)
2	50 (10%)

Abbreviations: ECOG PS: Eastern Cooperative Oncology Group performance status. ADL: activity of daily living, IADL: instrumental activity of daily living, VES-13: Vulnerable Elders Survey-13.

**Table 2:** Reasons for the first unplanned hospitalization in older patients with cancer treated with chemotherapy (*n* = 184).

Reported Cause	*n* (%)
Cancer related	53 (29)
Chemotherapy toxicity	37 (19)
Lung infection	30 (16)
Other infections	22 (12)
Falls and fractures	13 (7)
Cardiac events	9 (5)
Thromboembolic events	7 (4)
Neurological events	5 (3)
Hemorrhage/bleedings	4 (2)
Gastrointestinal events	4 (2)
Falls	2 (1)

**Table 3:** Factors associated with unplanned hospitalizations on univariable analysis.

Variable	UH (*n* = 184) *n*%	Non-UH (*n* = 309) *n*%	OR (95% CI)	*p* Value
Stage				
IV	118 (64)	166 (56)	1.54 (1.06–2.24)	0.024
I–III	66 (36)	143 (44)		
MAX2 index				
≥1	138 (75)	205 (66)	1.52 (1.02–2.29)	0.044
0	46 (25)	104 (34)		
Chemotherapy				
Standard therapy	98 (53)	117 (38)	1.91 (1.32–2.76)	0.001
Reduced/monotherapy	86 (47)	192 (62)		
Albumin g/dL				
≤3.5	51 (28)	30 (10)	3.61 (2.21–5.92)	<0.001
>3.5	133 (72)	279 (90)		
Creatinine Clearance mL/min				
<50	87 (47)	106 (34)	1.75 (1.20–2.54)	0.03
≥50	97 (53)	203 (66)		
Unintentional weight loss %				
>5%	80 (43)	69 (22)	2.67 (1.80–3.97)	<0.001
≤5%	104 (57)	240 (78)		
ECOG PS				
2	35 (19)	28 (9)	2.36 (1.38–4.03)	0.02
0–1	149 (81)	280 (91)		
Charlson comorbidity score				
≥2	84 (45)	101 (33)	1.73 (1.18–2.51)	0.004
0–1	100 (55)	208 (67)		
ADL				
≤5	52 (28)	54 (17)	1.87 (1.21–2.89)	0.005
6	132 (72)	255 (83)		
IADL				
≤7	87 (47)	187 (60)	1.71 (1.18–2.47)	0.004
8	97 (53)	122 (40)		
Falls in the past 6 months				
≥1	45 (24)	39 (13)	2.24 (1.39–3.60)	0.01
None	139 (76)	270 (87)		

Abbreviations: UH, unplanned hospitalization; ECOG PS, Eastern Cooperative Oncology Group performance status; ADL, activities of daily living; IADL: instrumental activities of daily living; Variables not reaching significance were age, sex, type of tumor, Short Physical Performance Battery (SPPB), body mass index, Pfeiffer test, Hospital Anxiety and Depression Scale (HDAS score), MOS Social Support Survey, Vulnerable Elders Survey-13 (VES-13), creatinine clearance, hemoglobin, and platelets and neutrophils counts.

**Table 4:** Variables significantly associated with unplanned hospitalizations on multivariable analysis.

Variable	β	SE	*p* †	OR (95% CI)	Score
Standard therapy	0.475	0.211	0.24	1.608 (1.064–2.431)	1
MAX2 index ≥ 1	0.558	0.215	0.02	1.748 (1.093–2.796)	1
Weight loss >5%	0.659	0.234	0.005	1.933 (1.222–3.059)	1
Albumin ≤ 3.5 g/dL	0.934	0.286	0.01	2.545 (1.453–4.456)	2
Charlson score ≥ 2	0.471	0.215	0.029	1.601 (1.050–2.440)	1
Falls in the past 6 months ≥ 1	0.614	0.268	0.022	1.847 (1.093–3.123)	1

CI = confidence interval; OR = odds ratio; † *p* values were calculated using a two-sided Wald test for multivariable analyses.

## Data Availability

De-identified individual data might be made available following publication by reasonable request to the corresponding author.

## Supplementary Materials

The following are available online at https://www.mdpi.com/2072-6694/13/6/1437/s1, Figure S1: Receiver operating characteristic (ROC) analyses to assess the capacity of the prognostic score to predict 6-month unplanned hospitalizations, Table S1: Summary of comprehensive geriatric assessment domains and elements.
