# Supplementary material for: Prediction of Unplanned Hospitalizations in Older Patients Treated with Chemotherapy
Source: Cancers (Basel). 2021 Mar 22;13(6):1437. doi: 10.3390/cancers13061437 (PMC8004134; doi:10.3390/cancers13061437)
Supplement: Supplementary file 1 [file cancers-13-01437-s001.pdf]

cancers

Supplementary Materials: Prediction of Unplanned Hospitalizations in Older Patients Treated with Chemotherapy

Jaime Feliu, Enrique Espinosa, Laura Basterretxea, Irene Paredero, Elisenda Llabrés, Beatriz Jiménez-Munárriz, Beatriz Losada, Alvaro Pinto, Ana Belén Custodio, María del Mar Muñoz, Jeniffer Gómez-Mediavilla, María Dolores Torregrosa, Patricia Cruz, Oliver Higuera and María José Molina-Garrido

Table S1. Summary of Comprehensive Geriatric Assessment Domains and Elements.

Domain	Elements of Assessment
Functional status	ECOG performance status [25]<br>Activities of daily living [29]<br>Instrumental activities of daily living [30]<br>Physical performance test SPPB [26]<br>Nº of falls in the last 6 months
Comorbidity	Charlson index [24]
Psychological status	Hospital Anxiety Scale [31]
Cognitive status	Pfeiffer test [28]
Social support	MOS Social Support Survey [32]
Nutritional status	Body mass index<br>Percent unintentional weight lost in the last 6 months

Cut off levels of the recommended geriatric assessment: Functional status by activities of daily living (ADL): independent score 6 versus dependent score ≤ 5. Instrumental activities of daily living (IADL): independent score 8/8 versus dependent score ≤7. Physical performance test SPPB: riesgo de fragilidad score <9/12. Presence of falls in the past 6 months: no falls versus at least one fall. Charlson index: 0-2 comorbidities versus comorbidities score ≥ 2, Hospital Hospital Anxiety and Depression Scale: Scores of 0 -7 in respective subscales are considered normal, with 8-10 borderline and 11 or over indicating clinical anxiety or depreesion. Pfeiffer test: ≥3/10 mistakes suggest cognitive impairment. MOS Social Support Survey 0-15/25 indica un apoyo social deficiente, y >15/25 un apoyo social adecuado.

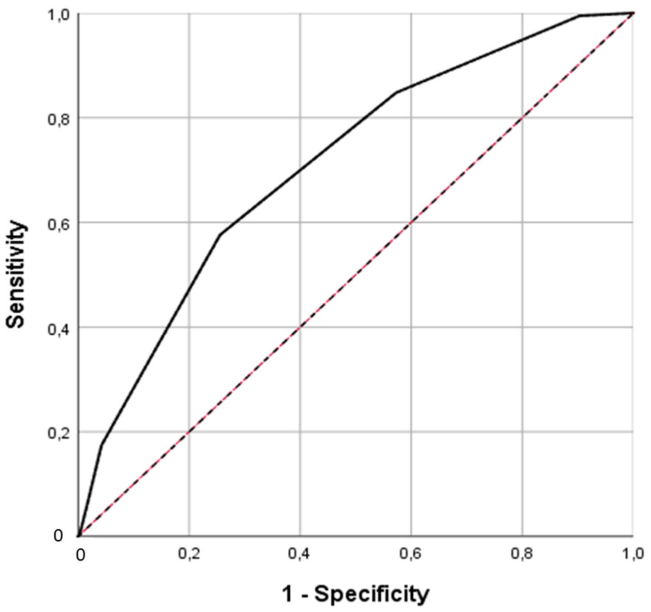

Figure S1. Receiver operating characteristic (ROC) analyses to assess the capacity of the prognostic score to predict 6-months unplanned hospitalizations.
